# Effects of embryo-derived exosomes on the development of bovine cloned embryos

**DOI:** 10.1371/journal.pone.0174535

**Published:** 2017-03-28

**Authors:** Pengxiang Qu, Suzhu Qing, Ruiqi Liu, Hongyu Qin, Weiwei Wang, Fang Qiao, Hui Ge, Jun Liu, Yong Zhang, Wei Cui, Yongsheng Wang

**Affiliations:** 1 College of Veterinary Medicine, Northwest A&F University, Key Laboratory of Animal Biotechnology of the Ministry of Agriculture, Yangling, Shaanxi, PR China; 2 Department of Veterinary and Animal Sciences, University of Massachusetts, Amherst, Massachusetts, United States of America; Qingdao Agricultural University, CHINA

## Abstract

The developmental competence of *in vitro* cultured (IVC) embryos is markedly lower than that of their *in vivo* counterparts, suggesting the need for optimization of IVC protocols. Embryo culture medium is routinely replaced three days after initial culture in bovine, however, whether this protocol is superior to continuous nonrenewal culture method under current conditions remains unclear. Using bovine somatic cell nuclear transfer (SCNT) embryos as the model, our results showed that compared with routine renewal treatment, nonrenewal culture system significantly improved blastocyst formation, blastocyst quality (increased total cell number, decreased stress and apoptosis, enhanced *Oct-4* expression and ratio of ICM/TE), as well as following development to term. Existence and function of SCNT embryo-derived exosomes were then investigated to reveal the cause of impaired development induced by culture medium replacement. Exosomes were successfully isolated through differential centrifugation and identified by both electron microscopy and immunostaining against exosomal membrane marker CD9. Supplementation of extracted exosomes into freshly renewed medium significantly rescued not only blastocyst formation and quality (*in vitro* development), but also following growth to term (*in vivo* development). Notably, ratio of ICM/TE and calving rate were enhanced to a similar level as that in nonrenewal group. In conclusion, our results for the first time indicate that 1: bovine SCNT embryos can secrete exosomes into chemically defined culture medium during IVC; 2: secreted exosomes are essential for SCNT blastocyst formation, blastocyst quality, and following development to term; 3: removal of exosomes induced by culture medium replacement impairs SCNT embryo development, which can be avoided by nonrenewal culture procedure or markedly recovered by exosome supplementation.

## Introduction

Embryos produced *in vitro* are generally inferior in quality and have lower developmental competence compared with their *in vivo* counterparts [1, 2]. Embryos produced *in vitro* from most mammalian species usually display fewer blastocysts capable of hatching and implantation, altered inner cell mass/trophectoderm (ICM/TE) ratios, higher apoptosis index, and lower total cell numbers. Moreover, these embryos present blastomeres with irregular sizes, increased sensitivity to cryopreservation, and abnormal gene expression and epigenetic modification [3–6]. Suboptimal culture conditions are main factors affecting the developmental competence of embryos produced *in vitro* [7]. Although various embryo culture conditions, such as culture medium, embryo density, supplementation of growth factors, and O_2_ concentration, have been extensively investigated and optimized [8–10], quality of embryos produced *in vitro* is still markedly lower, suggesting the involvement of other unknown factors that may be crucial for further optimization and improvement.

Replacing the culture medium is a routine step for *in vitro* culture (IVC) of bovine embryos. This process might have positive effects on the development of embryos by supplying some necessary nutrients for development and removing toxic metabolites, such as ammonia and free oxygen radicals accumulated in the culture medium during IVC [11, 12]. However, replacing the culture medium may also cause several negative effects. This process produces stress to the embryos, and a sudden change in the microenvironment might cause serious stress response for the embryos during IVC. Thus, comprehensive and unbiased evaluation of the impact of culture medium replacement on embryo development is important for optimization of embryo *in vitro* production. In the bovine, embryo culture medium is routinely replaced three days after initial culture [13].

Accumulating evidence has shown that preimplantation embryos can produce various paracrine factors, such as epidermal growth factor (EGF), platelet-activating factors, insulin-like growth factors, as well as messenger RNAs (mRNAs) and microRNAs (miRNAs). These paracrine factors have been reported to be essential for early embryonic development [14–16]. Several studies have shown that these paracrine factors are packaged into CD9 positive membranous micro-vesicles called exosomes, which are secreted or absorbed by the preimplantaion embryos through exocytosis or endocytosis, respectively [17, 18]. Therefore, replacing the medium during embryo culture also removes these essential exosomes and may reduce the developmental potential of embryos.

Somatic cell nuclear transfer (SCNT) technology is expected to be useful for animal breeding and research, but its efficiency of bovine cloning still remains low, and suboptimal culture system is one major reason [19, 20]. To optimize the *in vitro* culture system, we aimed to investigate whether SCNT embryos secrete exosomes, and the effects of these exosomes on development of cloned embryos. As previous studies have confirmed that exosomes exist in serum [21, 22], we applied the basic culture medium without serum [23] in our experiments.

Based on above-mentioned context, the present study was performed to determine the effects of culture medium replacement on developmental competence of bovine SCNT embryos. In addition, SCNT embryo-secreted exosomes were supplemented into freshly renewed medium to explore the effects on the developmental competence of SCNT embryos themselves.

## Materials and methods

This study was carried out in accordance with the guidelines for the care and use of animals of Northwest A&F University. All animal experimental procedures were approved by Animal Care Commission of College of Veterinary Medicine, Northwest A&F University. All effort has been made to minimize animal pain and suffering, and the cows and calves in the experiments are currently still alive and healthy in Keyuan Cloning Company, Shaanxi, China.

All chemicals were purchased from Sigma-Aldrich (St. Louis, MO, USA) unless otherwise noted. Disposable, sterile plasticware was purchased from Nunclon (Roskilde, Denmark).

### Oocyte collection and In Vitro Maturation (IVM)

Oocyte collection and IVM were performed as previously described [24]. Briefly, bovine cumulus–oocyte complexes (COCs) were aspirated from 2 mm to 8 mm antral follicles of ovaries obtained from Tumen abattoir in Xi’an, Shaanxi, China, and were washed thrice in phosphate-buffered saline (PBS) supplemented with 5% (v/v) fetal bovine serum (FBS). COCs with more than three layers of compact cumulus cells and uniform cytoplasm were used for IVM. Selected COCs were matured *in vitro* in bicarbonate-buffered tissue culture medium 199 (TCM-199, Gibco, BRL, Grand Island, NY, USA) supplemented with 10% (v/v) FBS, 1 μg/mL 17β-estradiol, and 0.075 IU/mL human menopausal gonadotropin in humidified air with 5% CO_2_ at 38.5°C for 21 h.

### Production of SCNT embryos and in vitro culture

Somatic cell-cloned embryos were produced and cultured *in vitro* as described previously [25]. After maturation, the COCs were treated with 0.2% hyaluronidase in PBS to disperse the cumulus cells from the oocytes. Oocytes with an extruded first polar body and even cytoplasm were selected for SCNT and stained with 10 μg/mL Hoechst 33342 for 10 min. Enucleation was performed by aspirating the first polar body and a small amount of surrounding cytoplasm with a 20 μm (internal diameter) glass pipette in microdrops of PBS supplemented with 7.5 μg/mL cytochalasin B and 10% FBS under an inverted microscope equipped with manipulation systems (Narishige, Japan). The aspirated cytoplasm was expelled in another microdrop and was observed under UV radiation to confirm the successful enucleation. The disaggregated bovine fibroblasts were used as nuclear donor cell and transferred to the perivitelline space of enucleated oocytes. The oocyte–cell fusion was performed with a pair of platinum electrodes connected to the micromanipulator in microdrops of Zimmermann’s fusion medium, and a double electrical pulse of 35 V for 10 μs was applied for oocyte–cell fusion. Reconstructed embryos were kept in modified synthetic oviduct fluid with amino acids (mSOFaa) containing 5 μg/mL cytochalasin B for 2 h until activation. Then the embryos were activated in 5 μM ionomycin for 4 min, followed by 4 h exposure to 1.9 mM dimethynopyridine in mSOFaa. The embryos were washed twice with the defined medium [23] and cultured in drops of 50 μL of the defined medium in humidified atmosphere with 5% CO_2_ in air at 38.5°C (Thermo Forma, USA). Droplets (50 μL) of the defined medium were prepared in 35-mm cell culture dish under mineral oil and equilibrated for 2 h before the embryos were loaded (10 embryos/microdrop). The defined medium was a basic culture medium, containing myo-inositol, a combination of insulin, transferrin, and selenium (ITS), EGF, and polyvinyl alcohol (PVA), which was consistent with our previous report [23]. Culture medium was replaced by transferring embryos into fresh equilibrated defined medium droplets on the day according to the experimental design.

### Immunofluorescence

Immunofluorescence was operated as described in detail previously [24, 26]. The embryos were fixed in 4% (v/v) paraformaldehyde in PBS for 2 h at room temperature, permeabilized with 0.2% (v/v) Triton X-100 in PBS for 20 min at room temperature, and blocked in blocking liquid (Beyotime, P0102) overnight at 4°C. The embryos were incubated overnight with primary anti-CDX2 mouse monoclonal antibody (BioGenex, Inc., San Ramon, CA) at 1:200 dilution in blocking buffer. After incubation, the embryos were washed in 0.1% PBS-PVA and treated with secondary antibody of Alexa Fluor 555-labeled goat anti-mouse IgG (Beyotime, A0459) at 1:500 dilution in dilution solution (Beyotime, P0108) for 2h at room temperature. After the embryos were washed thrice in 0.1% PBS-PVA for 5 min each wash, nuclear labeling was performed with 4,6-diamidino-2-phenylindole hydrochloride (DAPI, Vysis Inc., Downers Grove, USA) for 3 min. After wash and mounting, slides were examined by epifluorescence using a Nikon Eclipse Ti-S microscope (Nikon, Tokyo, Japan). All images were captured using Nikon DS-Ri1 digital camera and saved in TIFF format. All nuclei were identified by their blue fluorescence, while nuclei of trophectoderm cells (CDX2 positive) also exhibited red appearance.

### Apoptosis detection

Apoptosis detection was operated as described in detail previously [24, 26]. Apoptotic index was evaluated by DeadEnd Fluorometric TUNEL System (Promega, Madison, WI) according to the instruction manual. Briefly, day 7 blastocysts were fixed in 4% paraformaldehyde for 2 h in room temperature, permeabilized in 0.5% Triton X-100 for 5 min, and incubated with FITC-conjugated dUTP and terminal deoxynucleotidyl transferase at 37°C for 1 h in the dark (hereafter, all manipulations were performed in the dark). The tailing reaction was terminated in 2× SSC (SSC: 0.15 mol/L sodium chloride and 0.015 mol/L sodium citrate) for 15 min. Then the embryos were incubated in PBS containing 25 μg/mL RNase A for 30 min. The positive control (samples treated with DNase I at 37°C for 20 min) and negative control (only incubation buffer without terminal deoxynucleotidyl transferase) were also performed in parallel. After wash and DAPI staining, embryos were then mounted on slides and observed under Carl Zeiss LSM 510 laser confocal scanning microscope.

### Quantitative real-time PCR (q-PCR)

Quantitative real-time PCR was operated as described in detail previously [26]. Total RNA was extracted from day 7 blastocysts using Cells-to-Signal^™^ Kit (Invitrogen, USA) according to the manufacturer’s protocol. cDNA was synthesized using PrimeScript^™^ RT Reagent Kit (TaKaRa, Japan) with a total volume of 20 μL (4 μL of 5×RT buffer, 1 μL of RT enzyme mix, 1 μL of oligo dT primer, 1 μL of random 6-mers, 1 μg of RNA, and up to 20 μL of RNase-free dH_2_O). The expression levels of the examined genes were quantified by quantitative real-time PCR on the CFX96 real-time PCR detection system (Bio-Rad) using SYBR Premix Ex Taq^™^II (TaKaRa, Japan). The primers for quantitative PCR were synthesized as previously reported (Table 1). Reactions were performed in Low Tube Strip (Bio-Rad, GB). Each reaction mixture (20 μL) contained 2 μL (approximately 100 ng) of cDNA template, 10 μL of SYBR^®^ Premix Ex TII (2×), 0.8 μL of each PCR forward and reverse primers (10 μM), and 6.4 μL of dH_2_O. Thermal cycling conditions were 95°C for 1min, followed by 40 PCR cycles for 5s at 95°C for DNA denaturation and 30s at 60°C for primer annealing and extension. The melting protocol was from 65°C to 95°C (increment: 0.5°C/5s). Transcripts of examined genes were quantified in triplicates and calculated relative to the transcription in every sample of the housekeeping genes, *β-actin*, and *H2A*.*2*. The specificity of qPCR reaction was confirmed by both single peaks in the melt curves and gel electrophoresis. Water as negative control replaced cDNA in the real-time reaction tubes. Approximately 20 embryos per group were processed in each replication. Experiments were repeated at least thrice.

**Table 1:** Primer list of q-PCR.

Gene	Sequence	Product Size (bp)	Tm (℃)	Gene bank accession No.
*β-actin*	F (5'- 3') AAGGACCTCTACG CCAACACG	255	60	AY141970
	R (5'- 3') GAAGCATTTGCGG TGGACGAT			
*H2A*.*2*	F (5'- 3') GAGGAGCTGAACAAGCTGTTG	144	60	BF076713
	R (5'- 3')TTGTGGTGGCTCTCAGTCTTC			
*Bip*	F (5'- 3') GCTATTGCTTATGGCCTGGA	167	60	NM_001075148.1
	R (5'- 3') CGCTGGTCAAAGTCTTCTCC			
*Bax*	F (5'- 3') TTTGCTTCAGGGTTTCATCC	246	60	NM_173894.1
	R (5'- 3') CAGTTGAAGTTGCCGTCAGA			
*Bcl-2*	F (5'- 3') ATGTGTGTGGAGAGCGTCAA	137	60	NM_001166486.1
	R (5'- 3') TACAGCTCCACAAAGGCGTC			
*Oct-4*	F (5'- 3') GAGAGGTCCAACGGAGAGTG	297	60	NM_174580
	R (5'- 3') ACATGAGGAGCCAGGGTAAG			

F: forward primer; R: reverse primer.

### Isolation, purification, and identification of exosomes

Exosomes were isolated and purified using a published protocol as described in detail previously [27, 28]. The defined medium (35–40 μL) in the 35mm cell culture dish was collected on day 3 of embryo culture and immediately stored at −80°C. When desired accumulation (10 mL) was achieved, the medium was subjected to differential centrifugation at 4°C (300×*g*, 10 min to remove cells; 2, 000×*g*, 10 min to remove dead cells; and 10, 000×*g*, 30 min to remove cell debris, macroparticles, and apoptotic bodies) in 29×104 mm centrifuge tubes (Beckman, Palo Alto, CA, USA). The supernatants were then ultracentrifuged at 100, 000× *g* for 70 min in 14×95 mm ultra-clear centrifuge tubes (Beckman). The pellets from a single sample were pooled, resuspended in PBS, and again centrifuged at 100, 000× *g* for 70 min. Each pellet was finally resuspended in 30 μL of the defined medium to supplement the renewed culture medium. Exosomes were identified using a published protocol [28]. Briefly, 7.5 μL of the pellet suspension was top loaded on 300-mesh grids and dried. The grids were stained in 2% uranyl acetate and visualized with an energy-filtering transmission electron microscope (Carl Zeiss Microscopy GmbH, Oberkochen, Germany) at 120 kV. We followed the published protocols to determine immunofluorescence [29]. Briefly, 7.5 μL of purified pellet protein was incubated with 5 μL of 4 μm aldehyde/sulfate latex beads (Life Technologies Corp., Grand Island, NY, USA) in a 30 μL final volume of PBS at room temperature for 15 min. PBS (170 μL) was then added, and the mixture was incubated in a test tube rotator for 2.5 h at room temperature. Then 22 μL of 1M glycine/PBS was added and mixed gently to block the unbound sites of the latex beads. Then the mixture was allowed to stand on the bench for 30 min at room temperature. The beads were pelleted by centrifugation at 1,500×*g* for 3 min at room temperature, washed twice with 1 mL of PBS/0.5% BSA. The exosomes–bead complexes were incubated with anti-CD9 (MEM-61, Thermo Fisher Scientific, Rockford, IL, USA) conjugated to phycoerythrin for 1 h at room temperature. A negative control antibody reaction was performed using normal mouse IgG. The labeled exosomes–bead complexes were again pelleted and washed twice and finally resuspended in 20 μL of PBS/0.5% BSA. The final complexes (10 μL) were spread on a microscope slide with a drop of Dakocytomation fluorescent mounting medium (Dako, Carpinteria, CA, USA), air-dried, cover-slipped, and sealed with nail polish. The slides were examined using a HAL 100 fluorescence microscope (Carl Zeiss Microscopy GmbH). Experiments were repeated at least thrice.

### Embryo transfer

Healthy local Red Angles cows from Keyuan Cloning Company at two to five years age were used as SCNT embryo recipients. Unbiased recipient mother selection, embryo transfer manipulation, and caring and nursing were performed for all different groups. Per one blastocyst was loaded in one 0.25-mL straw, and transported from the laboratory to the experimental farm in Hepes-buffered TCM199 supplemented with 10% FBS at 37°C within 1 h. The embryos were non-surgically transferred to the uterine horns of the recipients on day 7 of their natural estrus cycle. Pregnancy was detected using rectal palpation/ultrasonography at 40, 90 and 120 day of gestation.

### Experimental design

In experiment 1, the exosomes from the culture medium were isolated and identified. On day 3 of embryo culture, culture medium was collected and used to detect whether SCNT embryos secrete exosomes into the surrounding environment. In addition, isolated exosomes were supplemented into the renewed group after culture medium replacement on day 3 of culture. Different concentrations of isolated exosomes were evaluated to define the suitable concentration to be used. The effects of exosome supplementation on the developmental competence of SCNT embryos were assessed based on blastocyst formation rate.

In experiment 2, the effects of medium replacement and supplementary extract of exosomes on the developmental competence of SCNT embryos were systematically evaluated. After activation, embryos were allocated randomly to nonrenewed, renewed and supplemented groups. The culture medium in the nonrenewed group was not refreshed during the *in vitro* embryo culture. By contrast, the culture medium in the renewed group was renewed on day 3 of IVC. Based on experiment 1, the optimum quantity of exosomes was supplemented into freshly renewed defined medium in the supplemented group. The effects of medium replacement and exosome supplementation on the development of SCNT embryos were comprehensively evaluated in terms of blastocyst formation rate, total cell number, apoptotic index, ICM/TE ratio, expression of several important development/stress/apoptosis-related genes in day 7 blastocysts, as well as the following *in vivo* development to term.

### Statistical analysis

Experiments were repeated at least thrice, and each replicate was performed using oocytes matured on the same day to remove any batch effect of oocytes. All embryos were allocated randomly to each treatment group. Blastocyst formation rate was analyzed with χ^2^ test. The total cell number and apoptosis index were determined using blastocysts randomly selected from each group, which contained approximately 20 embryos each replicate. Statistical comparisons were analyzed by one-way ANOVA. The relative abundances of gene transcripts were established by testing the data for normality and equal variance using the Levene median test, ANOVA, and followed multiple pair wise comparisons using the Tukey’s test. Statistical analyses were conducted using the SPSS software package (SPSS Inc., Chicago, IL, USA). Data were expressed as mean ± S.E.M. and P<0.05 was considered statistically significant.

## Results

### Presence of exosomes in SCNT embryo culture medium and effect of various doses of exosome supplementation on in vitro development of SCNT embryos

**Fig 1:**
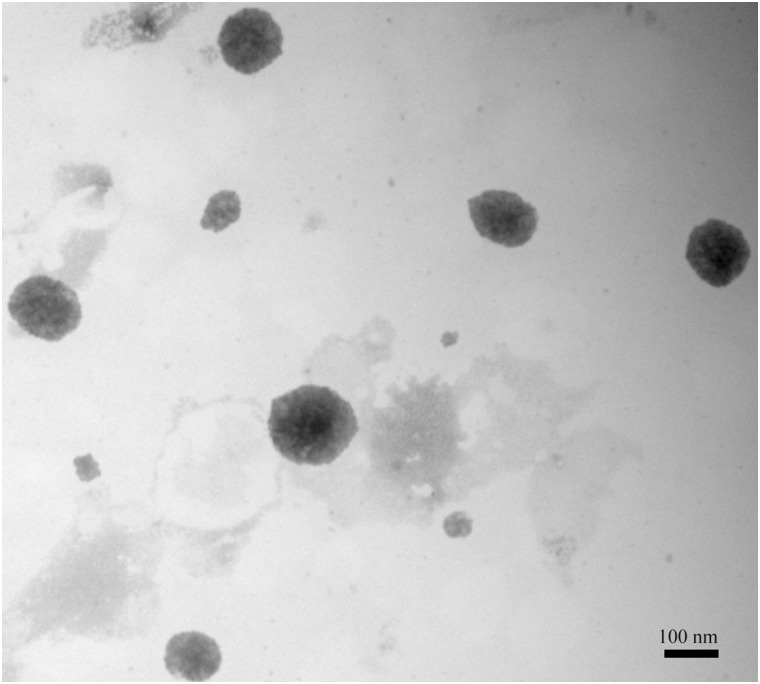
SCNT embryos-derived exosomes were identified by electron microscope analysis. Transmission Electron Microscopy (TEM) images showed the presence of exosomes after gradient centrifugations and negative staining with uranyl acetate. Bar, 100 nm.

**Fig 2:**
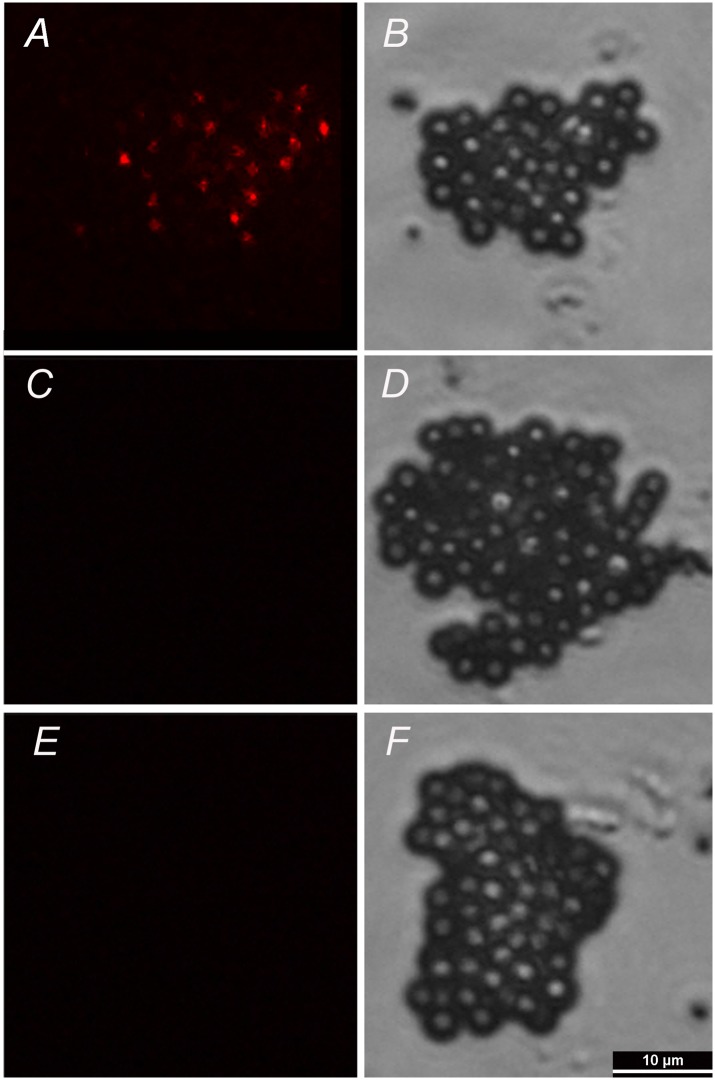
SCNT embryos-derived exosomes were identified by immunofluorescence. The exosomes were bound to beads of a size that was in the detection range of the fluorescence microscope (4-μm diameter latex beads). The beads were then bound to fluorescence-conjugated antibody against CD9. Images were taken under epifluorescence (A, C, E) and DIC (B, D, F). A,B: embryos-derived exosomes; C,D: IgG negative control; E,F: embryo-free culture medium control. Bar, 10 μm.

The exosomes in the embryo culture medium were isolated after several gradient centrifugation procedures. Electron microscopy results showed the presence of varied particles or spheres, with diameters mainly ranging from approximately 60 nm to 150 nm (Fig 1, S1 Table). The spheres were further characterized by immunostaining with specific antibody against exosomal membrane marker CD9. The results showed that strong and clear CD9+ signals were detected in spheres derived from embryo culture medium (Fig 2A and 2B), while no signal was identified in IgG negative control group (Fig 2C and 2D) or embryo-free culture medium control group (Fig 2E and 2F), indicating our observed signal was specific.

Effect of exosome supplementation on bovine SCNT embryo *in vitro* development was evaluated based on blastocyst formation rate. Exosomes extracted from 10 mL of day 3 culture medium were finally dissolved in 30 μL defined medium, of which 1, 5, and 10 μL were added into fresh medium during culture medium replacement at day 3 of embryo culture. Our results showed that whereas no obvious difference could be detected when 1 μL of exosome extract was added into freshly renewed culture medium (22.3% vs. 23.9%, P>0.05), addition of 5 μL and 10 μL both significantly improved blastocyst rate of bovine SCNT embryos (33.5% and 31.5%, respectively), with 5 μL group higher than 10 μL group but not significant (Table 2). Based on experimental design, dose of 5 μL exosome extract was adopted for the following experiments.

**Table 2:** Effect of various doses of exosome supplementation on blastocyst formation of *in vitro* cultured bovine SCNT embryos.

Added volume (μL)	No. embryo cultured	No. blastocyst (%)
0	206	46 (22.3) ^a^
1	201	48 (23.9) ^a^
5	194	65 (33.5) ^b^
10	181	57 (31.5) ^b^

^a, b^: different superscripts within same column indicate significant difference (P<0.05).

### Effect of culture medium renewal and optimal exosome supplementation on SCNT blastocyst quality and following in vivo development to term

**Fig 3:**
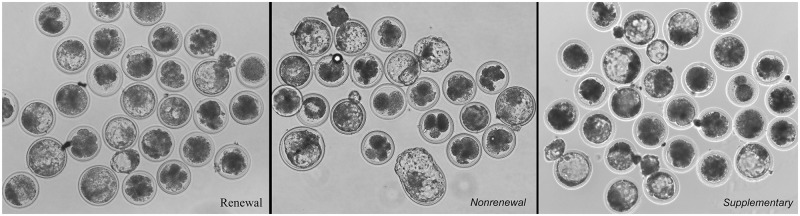
Representative photographs of bovine SCNT blastocysts derived from renewal, nonrenewal, and supplementary groups.

**Fig 4:**
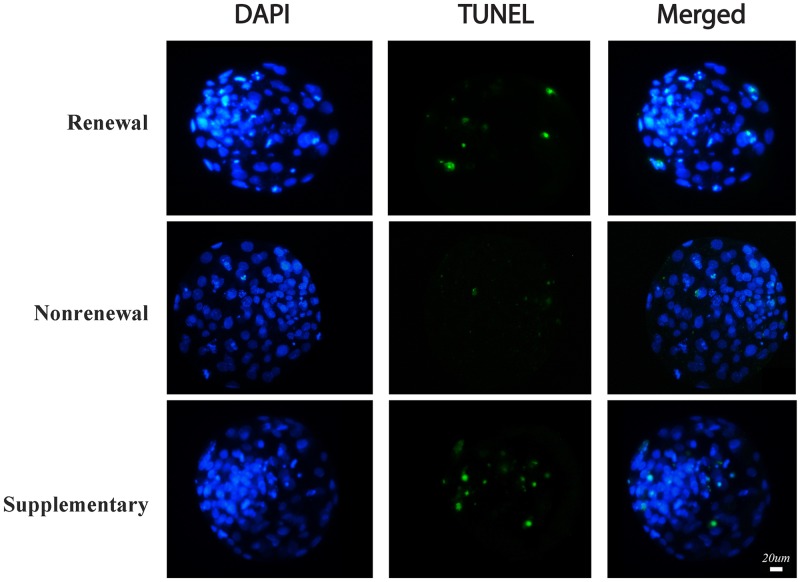
Apoptosis was assessed by TUNEL assay in bovine SCNT blastocysts derived from renewal, nonrenewal, and supplementary groups. The apoptotic blastomeres in day 7 blastocysts were detected by TUNEL (green). DNA was stained by DAPI (blue) to visualize all blastomeres. Bar, 20 μm.

**Fig 5:**
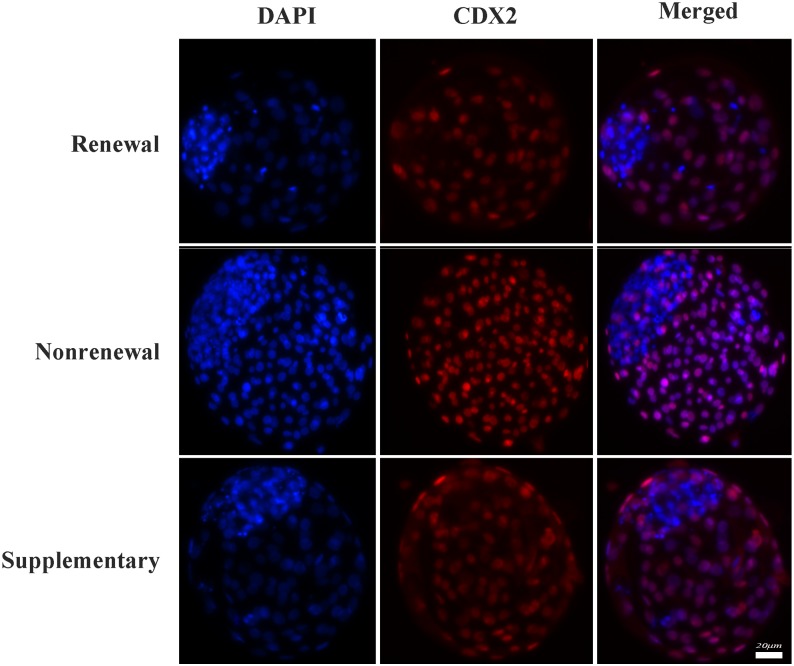
Ratio of ICM/TE in bovine SCNT blastocyst was assessed by immunostaining of CDX2, a specific marker of trophectoderm (red), as well as DAPI that stained nuclei of all blastomeres (blue). Bar, 20 μm.

**Fig 6:**
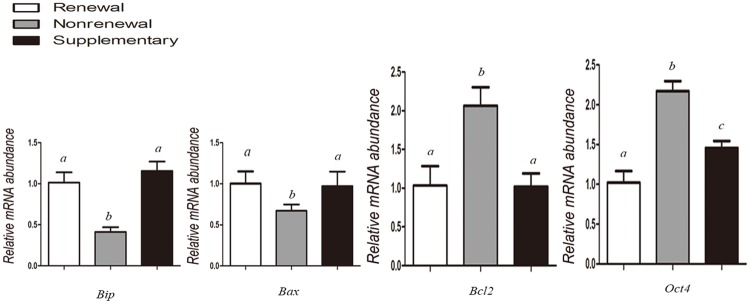
Relative expression levels of *Bip* (endoplasmic reticulum stress marker), *Bax* (pro-apoptosis marker), *Bcl-2* (anti-apoptosis marker), and *Oct-4* (pluripotency marker) were assessed by q-PCR in bovine SCNT blastocysts derived from renewal, nonrenewal, and supplementary groups. Values with different superscripts differ significantly (P<0.05).

Our results (Table 3) showed that compared with routine renewal treatment, nonrenewal treatment significantly improved blastocyst formation (37.8% vs. 23.1%, P<0.05) of SCNT embryos (Fig 3). Moreover, blastocysts derived from nonrenewal group exhibited higher total cell number (108.0 vs. 91.9, P<0.05), lower apoptotic index (1.9% vs. 6.3%, P<0.05) assessed by TUNEL (Fig 4, S2 Table) and higher ratio of ICM/TE (41.9% vs. 30.3%, P<0.05) assessed by immunofluorescence (Fig 5, S3 Table). In addition, expression levels of *Oct-4* (pluripotency marker) and *Bcl-2* (anti-apoptosis marker) were significantly higher, while *Bax* (pro-apoptosis marker) and *Bip* (endoplasmic reticulum stress marker) were significantly lower in the nonrenewal group than that of renewal group (Fig 6, S4 Table), indicating that culture medium replacement induced significant negative effects on *in vitro* development of bovine SCNT embryos.

**Table 3:** Effects of culture medium renewal and optimal exosome supplementation on *in vitro* development of bovine SCNT embryos.

Groups	No. embryo cultured	No. blastocyst (%)	No. blastomeres	Apoptotic index (%)	ICM/TE (%)
Renewal	412	95 (23.1) ^a^	91.9±5.4 ^a^	6.3±2.5 ^a^	30.3±3.9 ^a^
Nonrenewal	402	152 (37.8) ^b^	108.0±4.7 ^b^	1.9±1.6 ^b^	41.9±5.4 ^b^
Supplementary	402	124 (30.8) ^c^	99.1±5.6 ^c^	5.3±1.8 ^a^	39.5±3.7 ^b^

^a-c^: different superscripts within same column indicate significant difference (P<0.05).

Based on experimental design, 5 μL of exosome extract was added into freshly renewed culture medium to define roles of secreted exosomes in SCNT embryo development. The results (Table 3) showed that supplementary group had significantly higher blastocyst formation rate (30.8% vs. 23.1%, P<0.05), total cell numbers (99.1 vs. 91.9, P<0.05), ratio of ICM/TE (39.5% vs. 30.3%; P<0.05) (Fig 5), expression level of *Oct-4* (Fig 6) compared with routine renewal group, indicating exosomes secreted into culture medium are essential for embryo development and exosome supplementation can markedly rescue the impaired development induced by culture medium replacement. Among assessed markers of blastocyst quality, ratio of ICM/TE was enhanced to a similar level as that in nonrenewal group (Table 3), while with other markers compromised (Figs 4 and 6). These data demonstrated during *in vitro* culture of bovine SCNT preimplantation embryos, 1: secreted exosomes are functionally required for cell lineage specification (especially ICM allocation); 2: besides exosomes, other factors are also involved in preimplantation development, which will be disturbed by culture medium replacement.

To further evaluate effects of culture medium replacement and exosome supplementation on following *in vivo* development to term, blastocysts of each group were transferred into recipient cows. As shown in Table 4, significantly higher pregnancy rates (Day 40, 90 and 120) as well as full-term calving rates (S1 Fig) were observed in nonrenewal and supplementary groups than renewal group (P<0.05). In addition, there is no apparent difference between nonrenewal group and supplementary treatment (P>0.05). These results suggest culture medium replacement during *in vitro* culture of bovine SCNT preimplantation embryos impairs following *in vivo* development, which can be avoided by nonrenewal treatment, or recovered by exosome supplementation.

**Table 4:** Effect of culture medium renewal and optimal exosome supplementation on following *in vivo* development of bovine SCNT embryos after embryo transfer.

Groups	No.embryos transferred	No. of pregnancies (%)			Calves born (%)
		Day40	Day90	Day120	
Renewal	51	21 (41.2) ^a^	6 (11.8) ^a^	5 (9.8) ^a^	3 (5.9) ^a^
Nonrenewal	69	39 (56.5) ^b^	18 (26.1) ^b^	14 (20.3) ^b^	12 (17.4) ^b^
Supplementary	65	37 (56.9) ^b^	14 (21.5) ^b^	12 (18.5) ^b^	11 (16.9) ^b^

^a, b^: different superscripts within same column indicate significant difference (P<0.05).

## Discussion

Renewal of culture medium during *in vitro* culture (IVC) of preimplantation embryos has been adopted in many mammalian species, including human. However, whether this method is indeed superior to continuous nonrenewal culture remains unclear [30, 31]. Culture medium replacement may have positive effects on embryo development by supplying necessary nutrients and eliminating detrimental metabolites generated during culture, such as ammonia and free radicals derived from metabolic processes [11, 12]. However, renewal of culture medium also causes an abrupt change in the microenvironment. These sudden changes of physical and chemical properties may result in stress to cultured embryos [32]. In the bovine, embryo culture medium is routinely renewed three days after initial culture [13]. However, the effects of medium renewal on embryo development, especially, on SCNT embryo development, have not been extensively investigated. In this study, using bovine SCNT embryos as the model, we systematically assessed effects of culture medium renewal on blastocyst formation and quality (*in vitro* development) and following growth to term (*in vivo* development). Our results (Table 3) showed that compared with routine renewal treatment, continuous nonrenewal culture system exhibited a significant improvement in blastocyst formation, total cell number, ratio of ICM/TE (Fig 5), pregnancy and calving (Table 4), as well as a remarkable decrease in cellular stress (reduced expression of *Bip*, an endoplasmic reticulum stress marker [33], Fig 6) and apoptosis (increased *Bcl-2—*an anti-apoptosis marker [34] and reduced *Bax—*a pro-apoptosis marker [35], Fig 6; and less TUNEL signal, Fig 4). Notably, coupling of endoplasmic reticulum stress and apoptosis detected in our study is consistent with previous studies [36, 37]. Collectively, these results indicate that considering both *in vitro* and *in vivo* development of bovine SCNT embryos, continuous nonrenewal culture method is superior to routine renewal procedure under our current conditions.

Exosomes are small membrane vesicles that can be secreted from most cell types, including preimplantation embryos [18]. Exosomes contain not only proteins and lipids, but also mRNAs, miRNAs, and DNA cargoes and are thought to be essential for intercellular communications [38–41]. Exosome-mediated transfer of above-mentioned contents into other neighbor cells has been recently found indispensable for early embryo development [28, 42], implantation [29, 43] and pregnancy [18, 44]. Whereas existence and importance of exosomes have been confirmed in regular embryo development, very little is known in SCNT embryos. Our results for the first time demonstrate that exosomes can be secreted from bovine SCNT embryos, which were verified by both electron microscopy (Fig 1 and S1 Table) and immunostaining against exosomal membrane marker CD9 (Fig 2). To evaluate functions of exosomes in the development of bovine SCNT embryos, exosome supplementation experiments were performed. Our results showed that supplementation of exosomes into freshly renewed culture medium can significantly increase blastocyst formation rate, total cell numbers, ratio of ICM/TE (Table 3), expression of *Oct-4* (Fig 6), and calving rate (Table 4), indicating exosomes secreted into culture medium from SCNT embryos are essential for embryo development. However, exosome supplementation did not alter the expression of *Bax*, *Bcl-2*, *Bip* (Fig 6) or TUNEL assay result (Fig 4) compared with the medium renewal group (Table 3), suggesting only exosome addition cannot fully rescue or eliminate stress and apoptosis caused by culture medium replacement. Despite these results as discussed above, mechanism of the interaction between embryos and exosomes is still unclear and needs further investigation.

During preimplantation development, a crucial event is the first cell lineage specification: blastomeres located inside of the morula will give rise to the inner cell mass (ICM) from which the embryo is derived, while the outer blastomeres will differentiate exclusively into trophectoderm (TE) from which extra-embryonic tissues are derived [45, 46]. Meanwhile, differential gene expression patterns appear within distinct lineages. For example, mRNA of *Oct-4* (also known as *Pou5f1*) is solely enriched in ICM and functions to promote pluripotency and inhibit differentiation in these blastomeres [47, 48]. Previous studies in nuclear transfer embryos also confirmed that low expression of *Oct-4* at blastocyst stage would damage the following development [49–51]. In our study, mRNA of *Oct-4* was detected significantly higher in exosome supplementation group compared with medium renewal group (Fig 6), which may be the reason of higher ICM percentage in these blastocysts (Table 3). This finding is also consistent with a recent study in porcine embryos that demonstrated the existence of mRNAs of pluripotency genes (*Oct4*, *Sox2*, *Klf4*, *c-Myc*, and *Nanog*) in pig embryo-derived exosomes [28]. Interestingly, although exosome supplementation enhanced only ratio of ICM/TE to a similar level as that in nonrenewal group (Table 3), no overt difference in pregnancy or calving rate was detected between these two groups, suggesting ratio of ICM/TE during cell lineage specification might be a more critical marker than others for the following *in vivo* development to term. This suggestion actually also supports previous findings that satisfactory blastocyst rate of SCNT embryos did not guarantee the following *in vivo* development to term [49, 52].

Taken together, our experiments using bovine SCNT embryos as the model demonstrate for the first time that SCNT embryos also secrete exosomes into surrounding environment during IVC, which are essential for the following development of these embryos. Exosomes removal caused by culture medium replacement impairs both *in vitro* and *in vivo* development of SCNT embryos, which can be avoided by continuous nonrenewal culture or markedly rescued by exosome supplementation treatment.

## Supporting information

S1 Fig Cloned calves from renewal, nonrenewal and supplementary groups. (TIF)

S1 Table Size distribution of exosomes derived from embryo culture medium. (DOCX)

S2 Table Data of total cell number and apoptotic index in different groups. (XLSX)

S3 Table Data of ICM/TE ratio in different groups. (XLSX)

S4 Table Data of q-PCR in Bip, Bax, Bcl-2, and Oct-4. (DOC)
