# Supplementary figures and images for: Effects of embryo-derived exosomes on the development of bovine cloned embryos
Source: PLoS One. 2017 Mar 28;12(3):e0174535. doi: 10.1371/journal.pone.0174535 (PMC5370134; doi:10.1371/journal.pone.0174535)

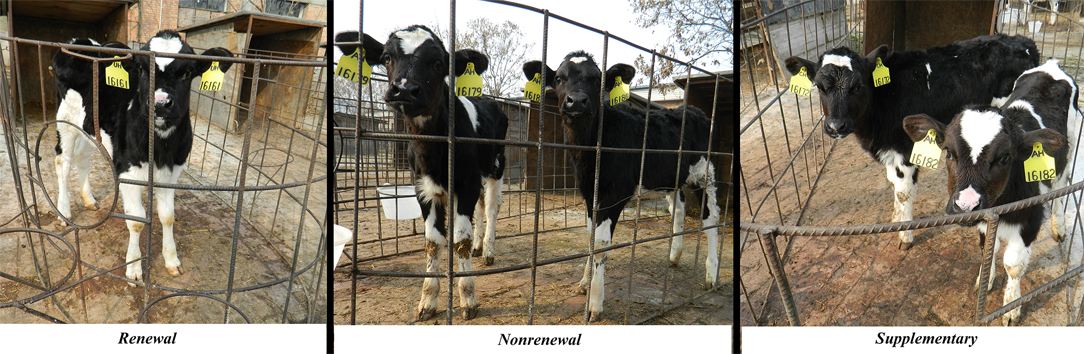

Supplement: S1 Fig — (TIF) [file pone.0174535.s001.tif]
