# Supplementary material for: Effects of embryo-derived exosomes on the development of bovine cloned embryos
Source: PLoS One. 2017 Mar 28;12(3):e0174535. doi: 10.1371/journal.pone.0174535 (PMC5370134; doi:10.1371/journal.pone.0174535)
Supplement: S1 Table — (DOCX) [file pone.0174535.s002.docx]

| Diameter (nm) | Number | Percentage of each group (%) |
| --- | --- | --- |
| 0＜exosomes≤30 | 14 | 1.95 |
| 30＜exosomes≤60 | 44 | 6.13 |
| 60＜exosomes≤90 | 127 | 17.71 |
| 90＜exosomes≤120 | 279 | 38.91 |
| 120＜exosomes≤150 | 161 | 22.45 |
| 150＜exosomes≤180 | 63 | 8.78 |
| 180＜exosomes≤210 | 29 | 4.04 |
| exosomes＞210 | 0 | 0 |

**S1 Table.** Size distribution of exosomes derived from embryo culture medium.
