# Supplementary material for: Effects of embryo-derived exosomes on the development of bovine cloned embryos
Source: PLoS One. 2017 Mar 28;12(3):e0174535. doi: 10.1371/journal.pone.0174535 (PMC5370134; doi:10.1371/journal.pone.0174535)
Supplement: S4 Table — (DOC) [file pone.0174535.s005.doc]

**S4 Table.** Data of q-PCR in *Bip*, *Bax*, *Bcl-2*, and *Oct-4*.

| Gene Symbol | Groups | Mean | SEM |
| --- | --- | --- | --- |
| *Bip* | Renewal | 1 | 0.12 |
|  | Nonrenewal | 0.41 | 0.06 |
|  | Supplementary | 1.16 | 0.11 |
| *Bax* | Renewal | 1 | 0.12 |
|  | Nonrenewal | 0.67 | 0.08 |
|  | Supplementary | 0.97 | 0.18 |
| *Bcl-2* | Renewal | 1 | 0.26 |
|  | Nonrenewal | *2.04* | 0.24 |
|  | Supplementary | *0.99* | 0.17 |
| *Oct-4* | Renewal | 1 | 0.15 |
|  | Nonrenewal | *2.16* | 0.13 |
|  | Supplementary | *1.45* | 0.09 |
